# The Identification of Marker Genes for Predicting the Osteogenic Differentiation Potential of Mesenchymal Stromal Cells

**DOI:** 10.3390/cimb43030150

**Published:** 2021-11-30

**Authors:** Masami Kanawa, Akira Igarashi, Katsumi Fujimoto, Tania Saskianti, Ayumu Nakashima, Yukihito Higashi, Hidemi Kurihara, Yukio Kato, Takeshi Kawamoto

**Affiliations:** 1 Natural Science Center for Basic Research and Development, Hiroshima University, Hiroshima 734-8533, Japan; mfuku@hiroshima-u.ac.jp; 2 Division of Advanced Technology and Development, BML, Inc., Saitama 350-1101, Japan; rasshi-a@bml.co.jp; 3 Department of Dental and Medical Biochemistry, Graduate School of Biomedical and Health Sciences, Hiroshima University, Hiroshima 734-8533, Japan; kfujimo@hiroshima-u.ac.jp (K.F.); tania-s@fkg.unair.ac.id (T.S.); yyykka@hotmail.co.jp (Y.K.); 4 Department of Molecular Biology and Biochemistry, Graduate School of Biomedical and Health Sciences, Hiroshima University, Hiroshima 734-8533, Japan; 5 Department of Pediatric Dentistry, Faculty of Dental Medicine, Universitas Airlangga, Surabaya 60132, Indonesia; 6 Department of Stem Cell Biology and Medicine, Graduate School of Biomedical and Health Sciences, Hiroshima University, Hiroshima 734-8533, Japan; ayumu@hiroshima-u.ac.jp; 7 Research Center for Radiation Genome Medicine, Research Institute for Radiation Biology and Medicine, Hiroshima University, Hiroshima 734-8533, Japan; yhigashi@hiroshima-u.ac.jp; 8 Department of Periodontal Medicine, Graduate School of Biomedical and Health Sciences, Hiroshima University, Hiroshima 734-8533, Japan; hkuri@hiroshima-u.ac.jp; 9 Writing Center, Hiroshima University, Higashi-Hiroshima 739-8512, Japan

**Keywords:** mesenchymal stromal cell, osteogenesis, fibroblast, predictive marker, ALP activity, regenerative medicine

## Abstract

Mesenchymal stromal cells (MSCs) have the potential to differentiate into a variety of mature cell types and are a promising source of regenerative medicine. The success of regenerative medicine using MSCs strongly depends on their differentiation potential. In this study, we sought to identify marker genes for predicting the osteogenic differentiation potential by comparing ilium MSC and fibroblast samples. We measured the mRNA levels of 95 candidate genes in nine ilium MSC and four fibroblast samples before osteogenic induction, and compared them with alkaline phosphatase (ALP) activity as a marker of osteogenic differentiation after induction. We identified 17 genes whose mRNA expression levels positively correlated with ALP activity. The chondrogenic and adipogenic differentiation potentials of jaw MSCs are much lower than those of ilium MSCs, although the osteogenic differentiation potential of jaw MSCs is comparable with that of ilium MSCs. To select markers suitable for predicting the osteogenic differentiation potential, we compared the mRNA levels of the 17 genes in ilium MSCs with those in jaw MSCs. The levels of 7 out of the 17 genes were not substantially different between the jaw and ilium MSCs, while the remaining 10 genes were expressed at significantly lower levels in jaw MSCs than in ilium MSCs. The mRNA levels of the seven similarly expressed genes were also compared with those in fibroblasts, which have little or no osteogenic differentiation potential. Among the seven genes, the mRNA levels of *IGF1* and *SRGN* in all MSCs examined were higher than those in any of the fibroblasts. These results suggest that measuring the mRNA levels of *IGF1* and *SRGN* before osteogenic induction will provide useful information for selecting competent MSCs for regenerative medicine, although the effectiveness of the markers is needed to be confirmed using a large number of MSCs, which have various levels of osteogenic differentiation potential.

## 1. Introduction

Mesenchymal stromal cells (MSCs) are multipotent precursor cells that differentiate into mature cells, such as osteocytes, adipocytes, chondrocytes, neurocytes, and cardiomyocytes [1,2,3]. Previous studies have reported the use of MSCs in regenerative medicine and tissue engineering [4]; in particular, MSCs would be applicable to clinical practice for a wide range of bone diseases, such as fracture nonunion and periodontal bone loss [5,6,7].

MSCs obtained from different tissues have differing differentiation abilities. For example, bone marrow-derived MSCs exhibit a higher osteogenic differentiation potential than adipose-derived MSCs [8]. In contrast, the adipogenic differentiation potential of adipose-derived MSCs is higher than that of bone marrow-derived MSCs, while MSCs derived from the synovium have a higher chondrogenic differentiation potential than bone marrow-derived MSCs [9]. Thus, MSCs obtained from different tissues seem to have intrinsic differentiation abilities related to their origin.

Herrmann et al. [10] reported that the chondrogenic differentiation potential of ilium MSCs was superior to that of tibia MSCs, although their osteogenic differentiation potentials were similar. In contrast, the chondrogenic and adipogenic differentiation potential of jaw MSCs were much lower than those of ilium MSCs, but the osteogenic differentiation potential of jaw MSCs was comparable to that of ilium MSCs [11,12]. Therefore, as the specific differentiation ability of MSCs depends on their source, it is highly desirable to assess the potential of MSCs to differentiate into the target tissue before their clinical application.

For the clinical application of MSCs, it is very important to check the quality of MSCs, such as the differentiation potential, before transplantation. Recent studies have shown that surface antigens and specific genes expressed in undifferentiated MSCs before differentiation induction can serve as markers to predict their differentiation potential. For example, CD271, CD146, and CD105 surface antigens have been reported as predictive markers of chondrogenic differentiation [13,14,15]. More recently, we identified predictive marker genes for chondrogenic and adipogenic differentiation of MSCs by taking advantage of the difference in differentiation abilities between ilium and jaw MSCs [16,17]. However, we could not identify predictive markers for their osteogenic differentiation potential by using the difference between ilium and jaw MSCs because there was no substantial difference. Although *WNT16*, the osteogenic differentiation predictive gene marker for tonsil-derived MSCs, was identified by Kim et al. [18], osteogenic differentiation predictive marker genes for bone marrow-derived MSCs have not yet been reported.

Fibroblasts are very similar to MSCs in terms of their origin and morphology [19]. Because there is little or no difference in the expression patterns of surface antigens between fibroblasts and MSCs, it is difficult to distinguish between these two cell types by assessing surface CD markers [19,20]. However, Igarashi et al. [12] successfully identified MSC marker genes that can distinguish between fibroblasts and MSCs by using differences in gene expression profiles between these cells. Of the 95 candidate genes selected with DNA microarrays, 9 were identified as MSC markers using the real-time quantitative polymerase chain reaction (RT-qPCR) for ilium, jaw, tibia, and femur MSCs, as well as fibroblasts. It has long been believed that fibroblasts have no differentiation potential [1]; however, Chen et al. [21] and Haniffa et al. [22] have recently reported that induced fibroblasts exhibit adipogenic, osteogenic, or chondrogenic differentiation phenotypes. Moreover, Fleury, et al. [23] found low, but significant, alkaline phosphatase (ALP) activity as an osteogenic differentiation marker after induction of fibroblasts. These findings suggest that osteogenic differentiation predictive markers can be identified by comparing osteogenic differentiation markers, such as ALP activity, with gene expression levels in fibroblasts and MSCs.

In this study, we sought to identify predictive markers of osteogenic differentiation for the selection of competent MSCs. Osteogenic differentiation was induced in nine ilium MSC samples and four fibroblast samples using osteogenic induction medium, and ALP activity was measured. We investigated the correlation between ALP activity in the 13 cell samples and the expression levels of 95 genes before induction. The results showed that there was a significant correlation between the expression levels of 17 genes and ALP activity. These genes can function as osteogenic predictive markers for the clinical application of MSCs.

## 2. Materials and Methods

### 2.1. Cells and Cell Culture

Bone marrow-derived MSCs and fibroblasts were obtained from patients admitted to Hiroshima University Hospital [12,16]. Fibroblasts were also obtained from Kurabo Industries (Osaka, Japan) [12]. The detailed information on cell donors is listed in Table S1. The detailed protocol for culture was described previously by Igarashi et al. [12]. Expression of cell surface molecules (CD105, CD73, CD90, CD34, CD14) were analyzed by flow cytometry as described (Table S2) [20]. By evaluating the differentiation markers, the osteogenic, chondrogenic, and adipogenic differentiation potentials were evaluated after inducing differentiation, as described previously (Table S2) [12,16]. The passage numbers of MSC and fibroblast cultures used for RT-qPCR and osteogenic differentiation analyses were shown in Table S1 [12]. All processes were performed after approval by the Ethics Committee of Hiroshima University (D-88-4). Written informed consent was obtained from all patients.

### 2.2. Osteogenic Differentiation

MSCs and fibroblasts were induced for osteogenic differentiation according to previous studies [12,24]. Briefly, 3 × 10^3^ cells per cm^2^ were seeded in a 24-well plate and grown until confluence. Confluent cells were maintained in osteogenic induction medium for 14 days. ALP activity of the induced cells was measured [25], and the values were normalized using DNA content [12].

### 2.3. RT-qPCR

RT-qPCR analysis of 95 candidate genes was performed using the ABI Prism 7900 Sequence Detection System with TaqMan (Applied Biosystems, Foster, CA, USA), as described previously [12]. mRNA expression levels were normalized to the mRNA level of β-actin. The TaqMan probe set IDs are listed in Table S3.

### 2.4. Statistical Analyses

All statistical analyses were performed using SPSS version 24 (IBM Corp., Armonk, NY, USA). Pearson’s correlation coefficient was calculated to analyze the correlation between gene expression levels and ALP activity. Gene expression levels among the three groups were evaluated using the Mann-Whitney U test.

## 3. Results

### 3.1. Selection of Candidate Genes for Osteogenic Predictive Markers

To identify candidates for osteogenic differentiation predictive markers, we examined the correlation between gene expression levels before osteogenic induction of ilium MSCs and the extent of MSC differentiation after induction. First, we measured the mRNA expression levels of 95 candidate genes in nine MSC and four fibroblast samples before induction (Table S3). After inducing osteogenic differentiation of these cells, we evaluated ALP activity on day 14 as an osteogenic differentiation marker. Correlation coefficients between the mRNA levels before induction and ALP activity after induction in the 13 cell samples were calculated. The results indicated that 17 out of 95 genes showed a significant positive correlation with ALP activity (Table 1). Thus, we identified 17 candidate predictive marker genes for the osteogenic potential of MSCs.

### 3.2. Comparison of Expression Levels of Candidate Genes for Osteogenic Predictive Markers among Three Different Cell Sources

**Figure 1:**
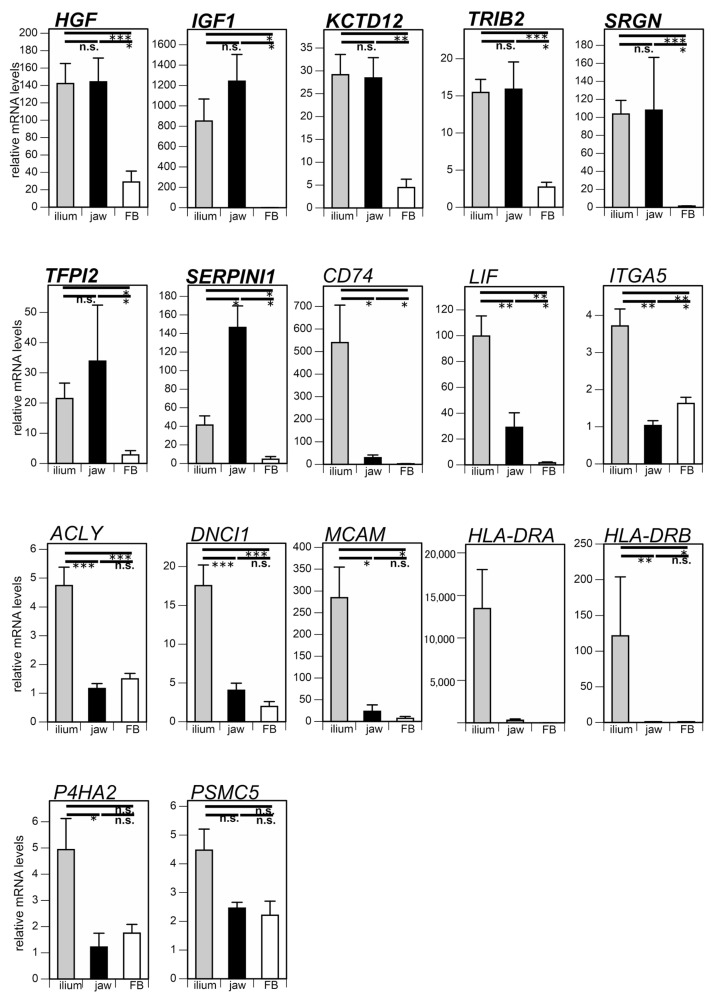
Comparison of osteogenic differentiation predictive markers among ilium mesenchymal stromal cells (MSCs) (*n* = 9), jaw MSCs (*n* = 5), and fibroblasts (FBs) (*n* = 4). mRNA levels of 17 marker genes were quantified before differentiation induction. The significance of differences between groups was analyzed by the Mann-Whitney U-test (* *p* < 0.05; ** *p* < 0.01; *** *p* < 0.001; n.s., not significant). Data are shown as means ± SEM.

Previous studies have shown that the chondrogenic and adipogenic differentiation potentials of jaw MSCs are much lower than those of ilium MSCs, but the osteogenic differentiation potential of jaw MSCs is comparable with that of ilium MSCs [11,12]. Therefore, osteogenic differentiation predictive markers are expected to be expressed in jaw and ilium MSCs at similar levels. In addition, marker genes should be expressed at much lower levels in fibroblasts. We compared mRNA levels of the 17 candidate genes among the ilium and jaw MSCs, and fibroblasts (Figure 1). The mRNA expression levels of *HGF*, *IGF*, *KCTD12*, *TRIB2*, *SRGN*, and *TFPI2* in jaw MSCs were similar to those in ilium MSCs. The mRNA level of *SERPINI1* in jaw MSCs was higher than that in ilium MSCs. In contrast, the expression levels of *CD74*, *LIF*, *ITGA5*, *ACLY*, *DNCI1*, *MCAM*, *HLA-DRA*, *HLA-DRB*, and *P4HA2* in jaw MSCs were much lower than those in ilium MSCs. Together, these results suggest that the seven genes, *HGF*, *IGF*, *KCTD12*, *TRIB2*, *SRGN*, *TFPI2*, and *SERPINI1*, can serve as osteogenic differentiation predictive markers. Importantly, the expression levels of these seven genes in both MSCs were much higher than those in fibroblasts, though the expression level of *PSMC5* was not different among ilium MSCs, jaw MSCs, and fibroblasts.

### 3.3. Comparison of Expresson Levels of Osteogenic Predictive Markers in Individual MSCs and Fibroblasts

**Figure 2:**
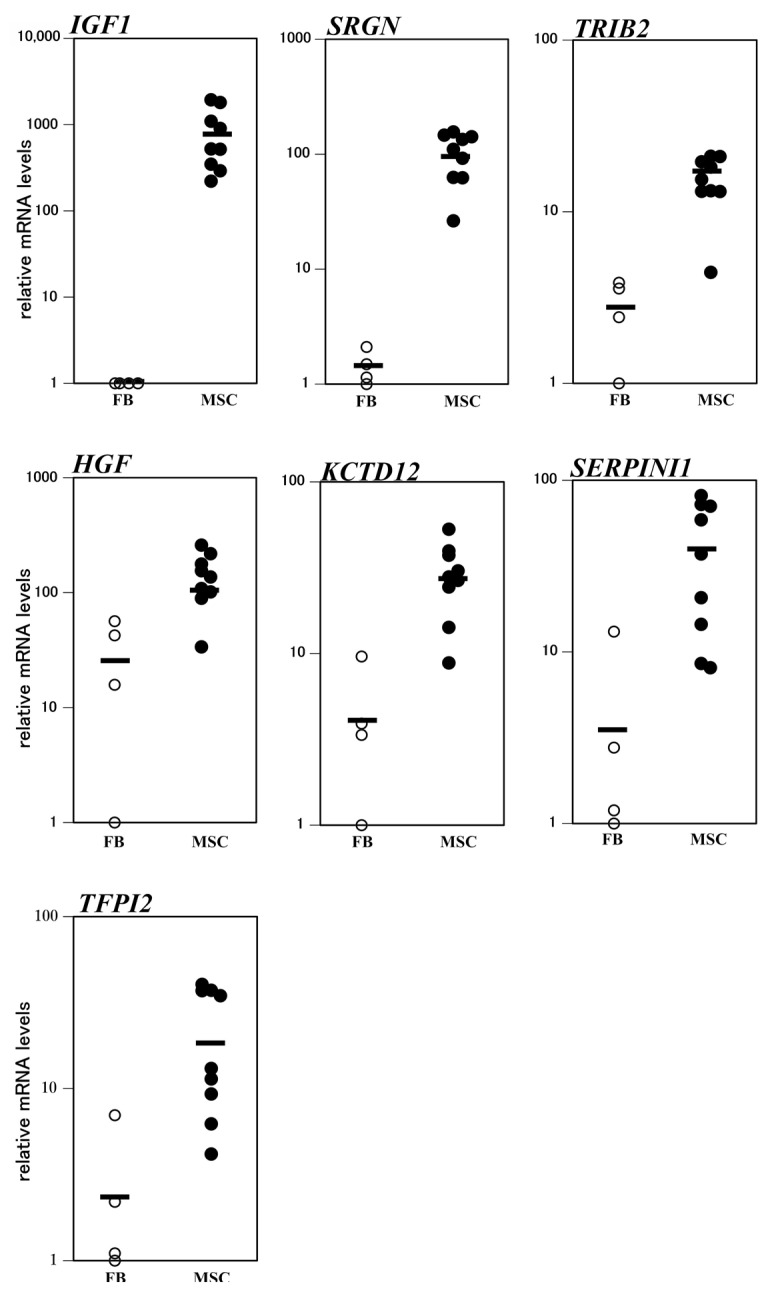
Analysis of mRNA levels of osteogenic differentiation predictive markers in individual fibroblasts (FBs) and ilium mesenchymal stromal cells (MSCs). The horizontal bars represent the mean value for each group.

Effective osteogenic differentiation predictive markers should be able to distinguish between fibroblasts and MSCs. To compare the expression levels of the seven osteogenic differentiation predictive marker genes among individual fibroblasts and MSCs, we analyzed their expression profiles in four individual fibroblast and nine individual MSC samples using scatter plots (Figure 2). The mRNA levels of *IGF1* and *SRGN* in all MSCs were higher than those in any of the examined fibroblasts. Namely, the minimum *IGF1* mRNA level in nine MSC samples was 221 times higher than the maximum value of those in the four fibroblast samples. Likewise, the minimum value of *SRGN* mRNA in the nine MSC samples was 12.5 times higher than the maximum value in the four fibroblast samples. Although the minimum value of *TRIB2* mRNA in the nine MSC samples was higher than the maximum value in the four fibroblast samples, the difference between them was marginal (1.1 times). Regarding *HGF*, *KCTD12*, *SERPINI1*, and *TFPI2*, the minimum mRNA values in the nine MSC samples were lower than the maximum values in the four fibroblast samples. Together, these results suggest that *IGF1* and *SRGN* are particularly potent osteogenic differentiation predictive markers.

### 3.4. Comparison of Differentiation Predictive Markers in Three MSC Lineages

In this study, we identified 17 candidate osteogenic prediction markers, which showed a significant correlation with the level of an osteogenic marker (ALP activity). Out of the 17 genes, 15 genes—*HGF*, *IGF1*, *KCTD12*, *TRIB2*, *SRGN*, *TFPI2*, *SERPINI1*, *CD74*, *LIF*, *ITGA5*, *ACLY*, *DNCI1*, *MCAM*, *HLA-DRA*, and *HLA-DRB*—were expressed at higher levels in ilium MSCs than in fibroblasts, although the levels of *P4HA2* and *PSMC5* in ilium MSCs were not significantly higher than those in fibroblasts. Then, we classified the 15 markers together with eight chondrogenic and 11 adipogenic predictive markers reported in our previous studies [16,17] using a Venn diagram. Among the 15 markers, *ACLY*, *CD74*, and *LIF* were identified as osteogenic, adipogenic, and chondrogenic predictive markers. In addition, *MCAM*, *DNCI1*, and *ITGA5* were recognized as osteogenic and adipogenic predictive markers, but not as chondrogenic predictive markers. The remaining nine genes—*HGF*, *SRGN*, *SERPINI1*, *TFPI2*, *KCTD12*, *TRIB2*, I*GF1*, *HLA-DRA*, and *HLA-DRB*—may serve only as osteogenic predictive markers. Thus, the 15 markers could be classified into three groups.

## 4. Discussion

In this study, we screened for osteogenic differentiation predictive markers of MSCs. The expression levels of 95 candidate genes in ilium bone marrow-derived MSCs and fibroblasts were compared with ALP activity as the differentiation marker. Of the 95 genes, the expression profiles of 17 correlated significantly with ALP activity. By comparing the mRNA levels of the 17 genes in jaw and ilium MSCs, we excluded 10 genes that showed much lower expression in jaw MSCs than in ilium MSCs. Thus, we identified seven genes as predictive markers for osteogenic differentiation. Among these seven genes, the mRNA levels of *IGF1* and *SRGN* in all MSCs were higher than those in any fibroblasts used in this study. Thus, these two genes may serve as effective markers for MSCs for use in bone regenerative therapy.

It is well known that *IGF1* plays an important role in bone formation by osteoblasts [26]. Recently, Koch et al. [27] showed that transfection with an IGF1-expressing adenovirus induced osteogenic marker genes, including type I collagen, Runx2, and ALP genes, in MSCs. This suggests that MSCs expressing high levels of *IGF1* have a high osteogenic differentiation potential. Accordingly, our results demonstrate that mRNA levels of *IGF1* in undifferentiated MSCs can predict the degree of osteogenic differentiation after induction.

SRGN is a proteoglycan with a repeated structure of Ser-Gly dipeptides. [28]. Bae et al. [29] reported that the expression level of *SRGN* in MSCs was 33.8 times higher than that in fibroblasts. In this study, *SRGN* expression in ilium MSCs was 72.5 times higher than that in fibroblasts (Figure 1). In addition, Kristensen et al. [30] found that the protein levels of SRGN in the culture supernatant of MSCs increased at the beginning of osteogenic induction. These findings suggest that SRGN mRNA and protein are highly expressed in MSCs before osteogenic induction, and SRGN is involved in osteogenic differentiation.

This study has several limitations. To select candidate osteogenic predictive markers, we used nine ilium MSCs and four fibroblasts as a model system consisting of cell lines, with various levels of differentiation potential. This system enabled us to select 17 candidate marker genes, which showed a significant correlation with osteogenic differentiation. However, when compared among nine ilium MSCs, the mRNA levels of *IGF1* and *SRGN* showed only weak correlations with the ALP activity as an osteogenic differentiation marker (r = 0.23 and r = 0.39, respectively) (Figure S1). The correlations were not statistically significant, which may be attributed to the small sample size of MSCs lines. In addition, we used MSCs with relatively high osteogenic differentiation potential in this study. To confirm the effectiveness of *IGF1* and *SRGN* as the osteogenic differentiation predictive markers, we would need to obtain low potential MSCs in the future investigation. Thus, further research is needed to assess the practical utility of markers obtained here using a large number of MSCs, which have different levels of osteogenic differentiation potential.

For clinical applications of MSCs in regenerative medicine, it is important to use MSCs without fibroblast contamination. To distinguish between MSCs and fibroblasts, Igarashi et al. [12] identified nine MSC marker genes. Six (*LIF*, *IGF1*, *SRGN*, *KCTD12*, *TRIB2*, and *DNCI1*) out of these nine MSC marker genes were included in the 15 genes identified in the present study. These results suggest that these six genes can serve not only as MSC markers, but also as osteogenic differentiation predictive markers. Bae et al. [29] also reported *SRGN* as a marker to discriminate MSCs from fibroblasts.

**Figure 3:**
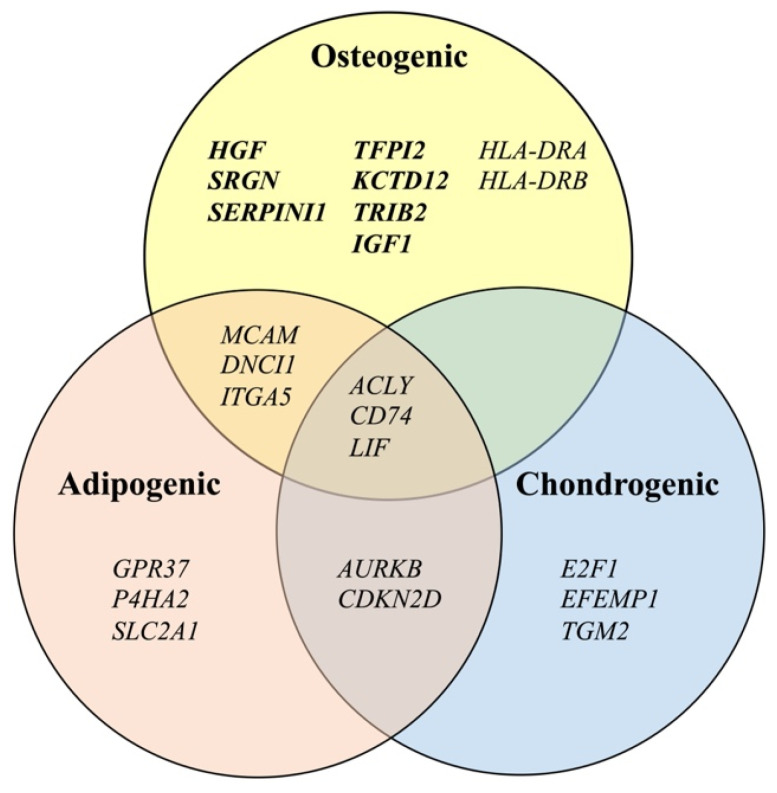
Classification of differentiation predictive marker genes for mesenchymal stromal cells into three lineages of osteogenic, adipogenic [17], and chondrogenic [16] differentiation.

As shown in Figure 3, the 15 osteogenic differentiation predictive markers identified in this study could be divided into three groups with different potentials: one lineage with osteogenic differentiation potential; two lineages with osteogenic and adipogenic differentiation potentials; and three lineages with osteogenic, adipogenic, and chondrogenic differentiation potentials. For bone-regenerative therapy, MSCs are not required to differentiate into all three lineages. In addition, it is important to control MSCs so that MSCs do not differentiate into other type of cells. Therefore, excellent markers for predicting the osteogenic differentiation potential can be powerful tools.

## 5. Conclusions

In this study, we identified two osteogenic differentiation predictive markers: *SRGN* and *IGF1*. This makes it possible to evaluate the differentiation potential of MSCs in an undifferentiated state as a quality evaluation of MSCs. These two markers will be useful to select MSCs suitable for bone regeneration and facilitate tissue engineering for bone diseases, such as fracture nonunion.

## Figures and Tables

**Table 1:** Osteogenic predictive marker genes that are significantly positively correlated with ALP activity after osteogenic induction.

Gene	Full Name	*R*
*MCAM*	melanoma cell adhesion molecule	0.889 **
*DNCI1*	dynein cytoplasmic 1 intermediate chain 1	0.824 **
*HGF*	hepatocyte growth factor	0.813 **
*HLA-DRA*	major histocompatibility complex, class II, DR alpha	0.780 **
*HLA-DRB*	major histocompatibility complex, class II, DR beta	0.762 **
*SRGN*	serglycin	0.727 **
*SERPINI1*	serpin family E member 1	0.724 **
*ACLY*	ATP citrate lyase	0.705 **
*P4HA2*	prolyl 4-hydroxylase subunit alpha 2	0.669 *
*ITGA5*	integrin subunit alpha5	0.648 *
*TFPI2*	tissue factor pathway inhibitor 2	0.620 *
*KCTD12*	potassium channel tetramerization domain containing 12	0.617 *
*LIF*	leukemia inhibitory factor	0.614 *
*PSMC5*	proteasome 26S Subunit, ATPase 5	0.589 *
*CD74*	CD74 molecule	0.585 *
*TRIB2*	tribbles pseudokinase 2	0.579 *
*IGF1*	Insulin-like growth factor 1	0.560 *

*R*: Pearson correlation coefficient; * *p* < 0.05, ** *p* < 0.01.

## Data Availability

The data presented in this study are available on request from the corresponding author.

## Supplementary Materials

The following are available online at https://www.mdpi.com/article/10.3390/cimb43030150/s1. Table S1: Donor information and passage numbers of cells used for RT-qPCR and differentiation analysis. Table S2: Characterization of cells used in this study. Table S3: Correlation between gene expression levels before osteogenic induction and ALP activities after induction of 13 cell lines (four fibroblast and nine MSC samples). Figure S1. Correlation between *IGF1* or *SRGN* mRNA levels before osteogenic induction and ALP activities after induction of nine ilium mesenchymal stromal cell samples.
