# Supplementary material for: The Identification of Marker Genes for Predicting the Osteogenic Differentiation Potential of Mesenchymal Stromal Cells
Source: Curr Issues Mol Biol. 2021 Nov 30;43(3):2157–66. doi: 10.3390/cimb43030150 (PMC8929155; doi:10.3390/cimb43030150)
Supplement: Supplementary file 1 [file cimb-43-00150-s001.zip › cimb-1377208-supplementary.pdf]

current issues in molecular biology

MDPI

Article

The Identification of Marker Genes for Predicting the Osteogenic Differentiation Potential of Mesenchymal Stromal Cells

Masami Kanawa [1], Akira Igarashi [2,3], Katsumi Fujimoto [3,4], Tania Saskianti [3,5], Ayumu Nakashima [6], Yukihito Higashi [7], Hidemi Kurihara [8], Yukio Kato [3] and Takeshi Kawamoto [3,9,*]

Table S1. Donor information and passage numbers of cells used for RT-qPCR and differentiation analysis.

donor ID	cell type	source	age	sex	disease/operation	passage number	
						RT-qPCR	differentiation
1	FB	skin	33	female	healthy	7	7
2	FB	skin	29	female	healthy	11	11
3	FB	skin	45	female	healthy	10	10
4	FB	gum	18	female	healthy	6	6
5	MSC	ilium	63	male	periodontitis	3	4
6	MSC	ilium	39	male	periodontitis	3	4
7	MSC	ilium	25	male	periodontitis	5	4
8	MSC	ilium	64	male	rebuilding of mandible	4	4
9	MSC	ilium	59	male	arteriosclerosis obliterans	4	4
10	MSC	ilium	55	male	arteriosclerosis obliterans	5	4
11	MSC	ilium	61	male	diabetes mellitus	5	4
12	MSC	ilium	53	male	Buerger's disease	3	4
13	MSC	ilium	81	male	arteriosclerosis obliterans	4	4
14	MSC	jaw	63	female	dental implant	4	4
15	MSC	jaw	36	male	fracture	5	4
16	MSC	jaw	36	female	fracture	5	4
17	MSC	jaw	20	male	jaw movement for orthodontic treatment	5	4
18	MSC	jaw	28	female	cyst removal	4	4

FB, fibroblasts; MSC, mesenchymal stromal cells.

Table S2. Characterization of cells used in this study.

donor ID	cell	CD marker					differentiation ability		
		CD105	CD73	CD90	CD34	CD14	OS	CH	AD
1	FB	+	+	+	−	−	±	−	−
2	FB	+	+	+	−	−	±	−	−
3	FB	+	+	+	−	−	±	−	−
4	FB	+	+	+	−	−	±	−	−
5	ilium MSC	+	+	+	−	−	+	+	+
6	ilium MSC	+	+	+	−	−	+	+	+
7	ilium MSC	+	+	+	−	−	+	+	+
8	ilium MSC	+	+	+	−	−	+	+	+
9	ilium MSC	+	+	+	−	−	+	+	+
10	ilium MSC	+	+	+	−	−	+	+	+
11	ilium MSC	+	+	+	−	−	+	+	+
12	ilium MSC	+	+	+	−	−	+	+	+
13	ilium MSC	+	+	+	−	−	+	+	+
14	jaw MSC	+	+	+	−	−	+	±	±
15	jaw MSC	+	+	+	−	−	+	±	±
16	jaw MSC	+	+	+	−	−	+	±	±
17	jaw MSC	+	+	+	−	−	+	±	±
18	jaw MSC	+	+	+	−	−	+	±	±

FB, fibroblasts; MSC, mesenchymal stromal cells. OS, osteogenic differentiation; CH, chondrogenic differentiation; AD, adipogenic differentiation.

Table S3. Correlation between gene expression levels before osteogenic induction and ALP activities after induction of 13 cell lines (four fibroblast and nine mesenchymal stromal cell samples).

donor ID number	1	2	3	4	5	6	7	8	9	10	11	12	13			
cell type	FB	FB	FB	FB	MSC	MSC	MSC	MSC	MSC	MSC	MSC	MSC	MSC	*r*	*p*	probe set ID
ALP (IU/μgDNA)	0.0205	0.0208	0.0251	0.0337	0.0681	0.1082	0.1118	0.1148	0.1182	0.1509	0.1624	0.1880	0.3219			
ACLY	1.00	1.49	1.91	1.60	2.40	1.02	6.06	3.86	6.04	6.07	5.59	6.30	5.35	0.705	0.007	Hs00153764_m1
ACVR2B	7.27	9.14	19.29	18.50	3.86	1.00	7.77	3.53	5.47	5.30	2.96	5.07	3.35	-0.561	0.046	Hs00609603_m1
ADD3	1.02	1.00	4.98	1.97	4.17	1.47	4.34	2.74	3.59	3.79	4.07	3.45	4.54	0.480	0.097	Hs00249890_m1
AMD1	2.26	2.07	5.25	4.02	2.05	1.00	5.35	3.25	3.93	3.95	4.34	6.22	4.66	0.411	0.163	Hs00750876_s1
AMFR	2.61	2.51	9.28	7.81	2.06	1.00	4.90	1.76	3.69	3.71	4.88	3.29	2.66	-0.293	0.332	Hs00181609_m1
AP1S2	8.67	5.26	44.81	28.05	1.53	1.00	7.97	2.45	2.65	4.22	2.62	5.35	4.93	-0.429	0.144	Hs00705223_s1
ARHGDIB	1.42	1.00	1.00	4.72	43.96	1.00	5.67	4.80	11.36	64.74	10.09	32.74	9.47	0.254	0.403	Hs00171288_m1
AURKB	3.38	2.76	1.38	1.45	1.00	1.12	4.18	5.28	5.24	7.25	1.44	10.68	1.68	0.260	0.391	Hs00177782_m1
AWP1	2.62	2.32	7.95	6.41	2.30	1.00	4.01	2.03	3.24	2.72	3.76	2.81	2.08	-0.382	0.197	Hs00180411_m1
BMI1	1.09	1.91	5.58	3.29	1.35	1.00	4.31	2.10	3.19	2.02	3.77	3.60	2.63	0.053	0.865	Hs00181626_m1
BMP4	1.00	1.00	43.19	1.00	30.52	45.76	633.96	164.10	210.38	42.89	239.19	234.85	135.54	0.325	0.279	Hs00259126_m1
CCNB1	2.10	1.49	2.76	2.93	1.00	1.03	4.68	5.07	4.05	6.51	2.10	8.81	2.18	0.306	0.310	Hs00277039_m1
CCND1	3.23	1.32	9.83	4.59	3.35	1.33	11.16	1.00	1.65	1.77	4.10	5.83	4.68	-0.007	0.983	Hs00269961_m1
CD74	3.35	1.00	3.85	1.00	374.54	210.65	169.91	339.70	440.14	1646.07	720.74	32.73	923.38	0.585	0.036	Hs00173542_m1
CD97	3.78	3.28	7.63	5.34	2.28	1.00	6.79	1.94	2.47	3.64	2.51	5.89	2.82	-0.224	0.461	Hs00415851_g1
CDC20	9.87	10.22	4.04	5.28	4.37	1.00	7.80	9.13	7.19	11.94	2.71	15.51	3.35	-0.038	0.902	Hs00176481_m1
CDKN2D	4.51	1.54	3.37	2.74	1.00	14.56	107.42	70.70	79.48	72.35	51.29	99.08	33.28	0.487	0.091	Hs00193192_m1
CDKN3	7.74	7.16	14.09	13.75	3.07	1.00	8.28	4.85	3.76	6.28	1.44	9.89	2.07	-0.498	0.084	Hs00164310_m1
COL7A1	1.59	3.64	4.66	4.33	1.49	1.00	6.75	2.09	2.20	2.79	4.62	4.92	1.76	-0.085	0.781	Hs00170014_m1
CTGF	3.60	1.56	2.62	1.00	57.34	17.51	19.45	30.32	25.20	17.51	35.88	31.88	32.32	0.520	0.069	Hs00189392_m1
DNCI1	1.00	3.73	1.13	2.01	13.74	4.26	15.91	9.07	19.25	21.94	18.98	29.97	24.94	0.824	0.001	Hs00181665_m1
DPYSL3	1.00	3.32	3.82	3.40	2.63	1.01	3.34	2.46	4.94	4.32	5.42	4.02	4.47	0.472	0.104	Hs00153451_m1
E2F1	3.72	1.83	2.60	2.14	1.74	1.00	5.83	4.82	4.16	7.37	2.56	7.44	2.75	0.305	0.311	Hs00173500_m1
EDG2	1.59	4.64	6.33	5.42	1.93	1.00	20.15	3.22	3.47	3.70	4.06	4.56	4.26	0.008	0.978	Hs00173857_m1
EDG7	1.42	13.71	61.53	1.90	1.00	3.44	371.40	1.00	4.57	1.00	4.51	1.00	3.32	-0.053	0.863	Hs00251661_m1
EFEMP1	1.00	1.00	1.00	1.00	5.06	5.28	3.15	10.34	8.41	24.85	12.75	6.85	3.99	0.373	0.210	Hs00154952_m1
EIF4G2	3.46	4.25	14.65	9.28	3.51	1.00	4.47	2.56	3.13	3.33	3.88	3.82	2.64	-0.447	0.126	Hs00169258_m1
F2R	1.08	70.49	34.43	18.55	13.19	1.58	40.85	5.28	2.03	1.00	3.98	2.05	3.42	-0.475	0.101	Hs00246260_m1
FLJ11175	1.00	5.03	9.57	5.05	2.30	3.93	75.94	21.06	48.35	4.10	28.14	28.73	23.13	0.325	0.278	Hs00181306_m1
FST	5.19	8.16	26.48	9.51	1.29	1.00	10.93	1.65	1.77	1.69	4.09	3.26	2.49	-0.450	0.123	Hs00178167_m1
GABRB1	1.00	1.00	1.00	1.00	1.00	1.00	1.00	1.00	1.00	1.00	1.00	1.00	1.00	-	-	Hs00173744_m1
GMFG	4.68	7.29	12.42	10.09	12.53	1.00	51.36	23.92	13.14	282.29	7.36	10.11	23.65	0.184	0.547	Hs00300159_m1
GPR37	152.36	1.00	436.84	139.04	1.00	11.43	22.59	21.54	25.68	25.67	33.71	55.00	22.46	-0.414	0.160	Hs00219578_m1
HGF	1.00	42.49	56.44	15.79	89.32	33.75	177.30	101.51	218.14	136.85	108.59	155.10	259.07	0.813	0.001	Hs00734212_m1
HLADRA	11.63	1.00	13.18	3.93	3581.96	3923.41	1850.79	6846.79	16390.07	31004.39	19316.54	182.51	38106.51	0.780	0.002	Hs00244919_m1
HLADRB	1.00	1.00	1.00	1.00	33.48	56.93	1.00	9.18	180.53	1.83	45.31	1.42	763.47	0.762	0.002	Hs00277001_m1
HNRPU	2.19	2.24	9.77	7.02	2.32	1.00	5.27	3.02	3.27	4.50	3.93	4.31	2.88	-0.223	0.463	Hs00197427_m1
ICAM1	2.04	4.22	3.90	4.30	11.14	1.00	3.65	3.82	25.14	9.41	16.11	3.31	9.05	0.266	0.380	Hs00153126_m1
IFI44	1.99	1.00	13.04	8.78	3.69	2.18	7.99	8.21	9.56	10.30	4.76	8.29	6.45	0.140	0.649	Hs00181213_m1
IGF1	1.00	1.00	1.00	1.00	291.96	523.55	221.12	905.25	1943.51	348.11	1809.53	519.23	1096.70	0.560	0.046	Hs00266026_m1
IGFBP5	1.00	30.96	2.91	3.67	19.85	5.40	33.86	5.26	8.50	35.09	12.83	21.81	24.98	0.378	0.203	Hs00152924_m1
IGFBP7	1.00	2.79	8.58	7.19	39.96	23.39	46.19	92.64	94.15	76.48	54.04	50.86	27.42	0.395	0.181	Hs00219878_m1
IL13RA2	21.97	2.68	250.30	31.42	1.00	2.43	55.24	7.20	3.25	13.19	28.86	61.36	78.01	-0.044	0.887	Hs00233722_m1
INPP5E	1.05	2.53	3.47	3.30	1.05	1.00	4.43	1.95	2.86	4.10	3.77	3.96	2.15	0.177	0.564	Hs00233743_m1
ITGA3	9.11	14.85	3.34	3.02	8.16	1.00	19.98	4.35	5.96	13.13	7.32	12.35	14.70	0.344	0.249	Hs00173952_m1
ITGA5	1.37	2.11	1.56	1.47	3.78	1.00	2.73	2.94	4.58	3.99	5.87	4.32	4.21	0.648	0.017	Hs00363726_m1
ITGA6	103.00	42.40	115.41	30.73	5.15	1.00	71.48	10.58	10.67	4.44	51.64	121.51	36.96	-0.126	0.682	Hs00222059_m1

KCNK12	1.00	1.00	3.96	1.00	1.00	1.00	1.00	1.00	1.00	1.00	1.00	1.00	1.00	-0.305	0.311	Hs00540818_s1
KCNN2	1.00	1.00	3.98	1.00	1.00	1.00	6.80	1.00	1.00	1.00	1.00	1.19	1.00	-0.134	0.661	Hs00165042_m1
KCTD12	1.00	3.91	9.61	3.36	26.68	8.82	53.00	14.20	39.63	30.33	24.41	27.94	37.40	0.617	0.025	Hs00174497_m1
LAMA3	1.16	5.72	1.00	1.05	6.29	19.63	56.60	13.88	27.28	7.92	49.34	23.02	27.70	0.522	0.067	Hs00171455_m1
LEPR	1.00	8.24	11.70	4.05	4.09	3.37	63.62	10.69	6.61	71.54	6.75	4.66	8.28	0.130	0.671	Hs00182998_m1
LIF	1.00	3.49	1.00	1.00	44.77	23.36	139.18	96.91	148.11	135.85	72.72	149.96	86.80	0.614	0.025	Hs00174838_m1
LRP8	2.01	3.95	4.10	2.11	2.01	1.00	8.67	2.04	4.55	2.00	5.16	8.21	4.42	0.331	0.269	Hs00198823_m1
MCAM	5.27	19.66	2.47	1.00	110.42	64.75	80.98	147.90	168.47	521.31	398.81	483.48	586.28	0.889	0.00005	Hs00428518_m1
MCM5	8.15	7.03	5.89	3.60	3.83	1.00	5.43	4.92	3.96	6.46	2.17	8.18	2.45	-0.330	0.270	Hs00217924_m1
MCM7	5.22	3.62	3.86	2.89	4.18	1.00	7.26	5.64	4.07	6.76	2.99	8.01	2.89	0.080	0.794	Hs00179899_m1
MGC5306	1.02	1.22	3.05	1.15	1.25	1.00	5.71	2.69	2.96	2.68	3.47	3.99	3.13	0.471	0.104	Hs00741286_m1
MGP	1.00	2.04	15.57	1.59	123.43	86.15	151.52	51.82	979.77	160.54	662.92	37.15	205.98	0.289	0.339	Hs00204417_m1
MICA	1.00	1.92	2.79	2.04	1.65	1.37	3.72	2.34	3.88	3.58	5.24	8.39	2.38	0.427	0.146	Hs00852569_g1
NDUFA8	4.44	3.47	16.43	11.58	2.92	1.00	4.19	2.04	3.62	4.38	3.52	5.21	3.79	-0.365	0.220	Hs00168558_m1
NFE2L3	5.22	6.15	14.59	9.28	9.18	1.00	8.92	5.51	6.59	20.47	3.37	9.88	6.81	-0.029	0.926	Hs00159686_m1
NPR3	1.43	6.75	10.09	1.00	96.75	97.94	187.65	212.87	84.96	640.36	99.33	137.09	128.03	0.372	0.211	Hs00188349_m1
NT5E	3.20	2.33	12.02	7.28	3.26	1.00	15.55	2.48	3.19	4.26	3.16	9.67	4.50	-0.040	0.898	Hs00172183_m1
P4HA2	1.00	1.50	2.58	1.91	2.02	2.18	1.38	2.87	8.12	1.51	9.48	9.22	7.65	0.669	0.012	Hs00175051_m1
PGR	1.44	4.49	19.40	4.67	1.00	1.39	73.41	1.00	4.12	2.24	1.31	4.51	4.31	-0.056	0.857	Hs00170182_m1
PGRMC2	5.33	5.34	18.52	16.75	3.88	1.00	6.58	2.87	3.51	3.63	5.31	3.31	3.60	-0.473	0.103	Hs00356977_m1
PLAU	1.00	897.17	347.23	333.23	3019.26	264.08	1160.75	916.28	1838.36	1259.14	1187.44	450.06	637.77	0.037	0.906	Hs00172888_m1
PLEC1	2.62	3.05	5.97	5.22	4.01	1.00	5.53	1.82	1.83	3.64	3.83	4.19	2.96	-0.192	0.530	Hs00160444_m1
PQBP1	1.00	3.69	21.81	23.43	3.18	1.00	5.95	3.47	3.71	4.98	4.29	5.01	3.22	-0.368	0.216	Hs00176952_m1
SRGN	1.14	2.10	1.00	1.49	146.54	26.33	62.83	62.36	92.16	156.23	134.47	110.28	141.90	0.727	0.005	Hs00165590_m1
PRKAG1	1.60	1.66	5.32	4.31	1.44	1.00	5.85	2.75	4.46	5.59	4.56	5.38	3.96	0.334	0.265	Hs00267687_m1
PROS1	1.13	1.00	8.99	5.20	2.35	5.71	14.51	12.68	23.04	9.81	23.04	8.82	8.62	0.380	0.200	Hs00267896_m1
PSMC5	1.48	1.30	3.34	2.72	1.00	1.19	5.76	3.66	5.14	6.34	5.32	7.44	4.44	0.589	0.034	Hs00357247_g1
RAB3B	2.93	6.57	16.05	11.08	1.00	1.23	40.65	3.43	3.17	1.94	4.94	9.24	5.77	-0.076	0.805	Hs00192380_m1
RRM2	1.95	1.76	1.80	1.28	1.00	0.74	4.47	4.46	4.12	6.12	1.53	6.78	1.55	0.281	0.352	Hs00190794_m1
SERPINI1	1.00	2.77	13.13	1.19	8.09	14.48	81.41	20.69	37.20	8.56	58.80	72.28	70.59	0.724	0.005	Hs00193673_m1
SLC16A4	1.00	6.50	5.26	3.34	6.27	2.06	16.80	4.47	5.92	14.34	6.81	6.77	9.12	0.389	0.189	Hs00197884_m1
SLC20A1	1.73	1.99	5.81	4.19	6.95	1.00	7.63	3.26	4.29	3.57	4.17	8.01	6.38	0.389	0.189	Hs00366488_m1
SLC2A1	1.00	2.89	1.28	1.34	7.48	1.23	4.44	4.58	6.90	7.42	5.87	6.55	4.16	0.456	0.117	Hs00183386_m1
SLCO1A2	1.00	1.00	1.00	1.00	1.00	1.00	1.00	1.00	1.00	1.00	9.29	1.00	1.00	0.182	0.552	Hs00225533_m1
SPRY2	1.16	1.17	8.43	3.07	2.00	1.00	7.02	1.88	2.39	4.36	2.45	3.96	2.31	-0.062	0.841	Hs00232525_m1
TCF19	6.10	3.67	3.10	2.65	2.97	1.00	3.68	3.58	3.21	4.09	1.79	7.14	1.98	-0.134	0.662	Hs00197918_m1
TFPI2	1.00	2.19	6.99	1.10	37.27	6.22	13.07	4.15	11.36	40.32	9.29	37.07	34.66	0.620	0.024	Hs00190278_m1
TGM2	1.00	6.93	2.94	1.40	398.85	38.40	100.31	166.17	314.09	555.61	148.05	114.79	101.72	0.253	0.404	Hs00165949_m1
TIMP3	1.00	1.22	15.39	11.10	2.27	2.73	3.24	3.45	3.65	1.16	6.23	4.72	3.52	-0.229	0.452	Hs00177406_m1
TK1	7.63	8.65	7.37	4.72	3.60	1.00	10.24	5.42	5.25	10.30	2.12	11.22	3.30	-0.146	0.635	Hs00222224_m1
TRIB2	2.43	1.00	3.57	3.86	13.15	4.43	19.54	15.42	21.05	20.97	13.11	18.21	13.25	0.579	0.038	Hs00188500_m1
TRIP13	2.73	2.24	4.11	3.60	1.00	1.11	5.92	5.18	4.13	6.74	2.36	8.28	2.15	0.179	0.559	Hs00853610_g1
UBE2C	2.08	2.03	2.13	1.39	1.11	1.00	4.94	7.90	5.72	7.87	2.11	10.38	2.40	0.343	0.251	Hs00819350_m1
UBE2S	4.67	8.37	4.93	5.76	5.83	7.43	3.31	3.95	5.82	1.00	10.16	0.078	5.27	0.078	0.801	Hs00188233_m1
UCHL1	3.42	17.51	1.71	1.00	6.68	6.53	5.78	8.27	5.30	1.40	11.40	0.428	9.46	0.428	0.144	Hs00182461_m1
VLDLR	9.15	6.90	9.17	3.40	4.56	6.91	5.65	3.50	3.29	1.00	6.26	0.034	22.63	0.034	0.912	Hs00183662_m1
WIF1	1.00	2.93	14.36	2.65	1.92	1.59	1.00	1.00	1.20	1.00	1.14	-0.330	1.68	-0.330	0.270	Hs00375275_m1
ZNF185	7.85	3.92	4.77	23.85	4.95	3.83	2.23	8.28	3.67	1.00	8.20	-0.189	5.68	-0.189	0.535	Hs00200253_m1

r, Pearson correlation coefficient; FB, fibroblasts; MSC, ilium mesenchymal stromal cells.

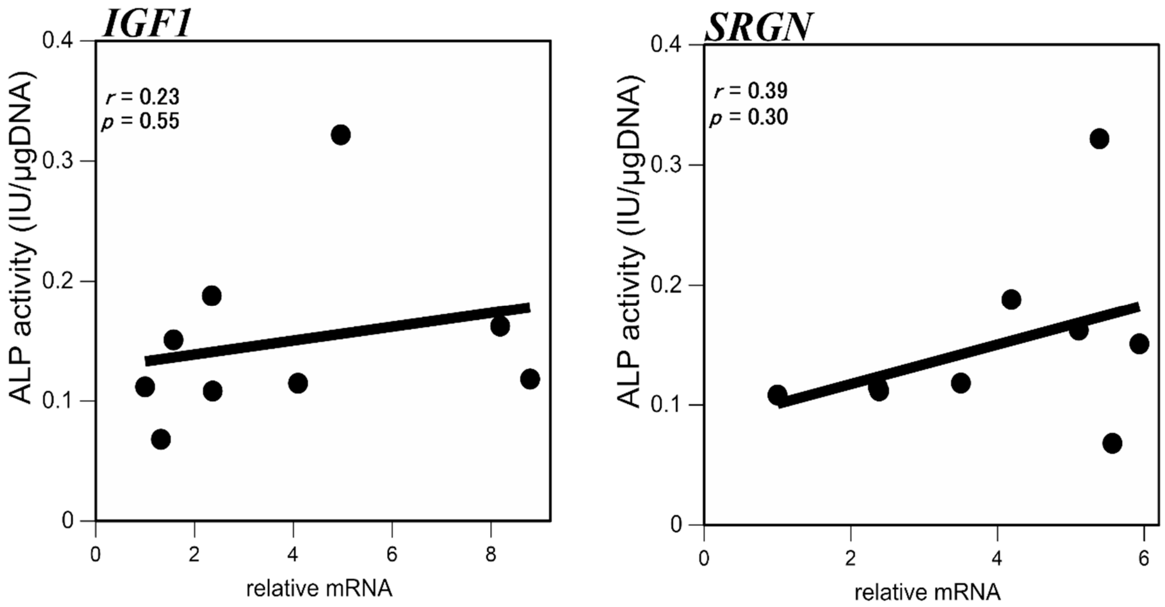

Figure S1. Correlation between *IGF1* or *SRGN* mRNA levels before osteogenic induction and ALP activities after induction of nine ilium mesenchymal stromal cell samples. *r*, Pearson correlation coefficient.
